# Fractional gradient optimized explainable convolutional neural network for Alzheimer's disease diagnosis

**DOI:** 10.1016/j.heliyon.2024.e39037

**Published:** 2024-10-09

**Authors:** Zeshan Aslam Khan, Muhammad Waqar, Naveed Ishtiaq Chaudhary, Muhammad Junaid Ali Asif Raja, Saadia Khan, Farrukh Aslam Khan, Iqra Ishtiaq Chaudhary, Muhammad Asif Zahoor Raja

**Affiliations:** a International Graduate School of Artificial Intelligence, National Yunlin University of Science and Technology, 123 University Road, Section 3, Douliou, Yunlin, 64002, Taiwan, Republic of China; b Future Technology Research Center, National Yunlin University of Science and Technology, 123 University Road, Section 3, Douliu, Yunlin, 64002, Taiwan, Republic of China; c Department of Computer Science and Information Engineering, National Yunlin University of Science and Technology, 123 University Road, Section 3, Douliou, Yunlin, 64002, Taiwan, Republic of China; d Medicare Hospital, Saidpur Road, Rawalpindi 46000, Pakistan; e Center of Excellence in Information Assurance, King Saud University, Riyadh, 11653, Saudi Arabia; f FOCAS Research Institute, Technological University Dublin, Dublin, Ireland

**Keywords:** Alzheimer's disease, Convolutional neural network, Fractional optimization, Neuroimaging, Explainable artificial intelligence, Customized pooling

## Abstract

Alzheimer's is one of the brain syndromes that steadily affects the brain memory. The early stage of Alzheimer's disease (AD) is referred to as mild cognitive impairment (MCI), and the growth of Alzheimer's is not certain in patients with MCI. The premature detection of Alzheimer's is crucial for maintaining healthy brain function and avoiding memory loss. Different multi-neural network architectures have been proposed by researchers for efficient and accurate AD detection. The absence of improved feature extraction mechanisms and unexplored efficient optimizers in complex benchmark architectures lead to an inefficient and inaccurate AD classification. Moreover, the standard convolutional neural network (CNN)-based architectures for Alzheimer's diagnosis lack interpretability in their predictions. An interpretable, simplified, yet effective deep learning model is required for the accurate classification of AD. In this study, a generalized fractional order-based CNN classifier with explainable artificial intelligence (XAI) capabilities is proposed for accurate, efficient, and interpretable classification of AD diagnosis. The proposed study (a) classifies AD accurately by incorporating unexplored pooling technique with enhanced feature extraction mechanism, (b) provides fractional order-based optimization approach for adaptive learning and fast convergence speed, and (c) suggests an interpretable method for proving the transparency of the model. The proposed model outperforms complex benchmark architectures with regard to accuracy using standard ADNI dataset. The proposed fractional order-based CNN classifier achieves an improved accuracy of 99 % as compared to the state-of-the-art models.

**Table:** Nomenclature

Abbreviation	Full Form
DL	Deep Learning
AD	Alzheimer's Disease
MCI	Mild Cognitive Impairment
Fr-CNN	Fractional Order Convolutional Neural Network
XAI	Explainable Artificial Intelligence
ADNI	Alzheimer's Disease Neuroimaging Initiative
LIME	Local Interpretable Model-Agnostic Explanations
FSGD	Fractional Stochastic Gradient Descent
GFSGD	Generalized Fractional Stochastic Gradient Descent
MRI	Magnetic Resonance Imaging
SVM	Support Vector Machine
KNN	K-Nearest Neighbors
VGG	Visual Geometry Group (a deep learning model)
ADASYN	Adaptive Synthetic Sampling (an oversampling technique)
FLOPs	Floating Point Operations
PCA	Principal Component Analysis
ICA	Independent Component Analysis
3D-CNN	3-Dimensional Convolutional Neural Network
EEG	Electroencephalogram
DWT	Discrete Wavelet Transform
EMD	Empirical Mode Decomposition
NCSA	Non-Local Context Spatial Attention
SOTA	State of the Art
Alpha (α)	Fractional Order

## 1 Introduction

**Fig. 1:**
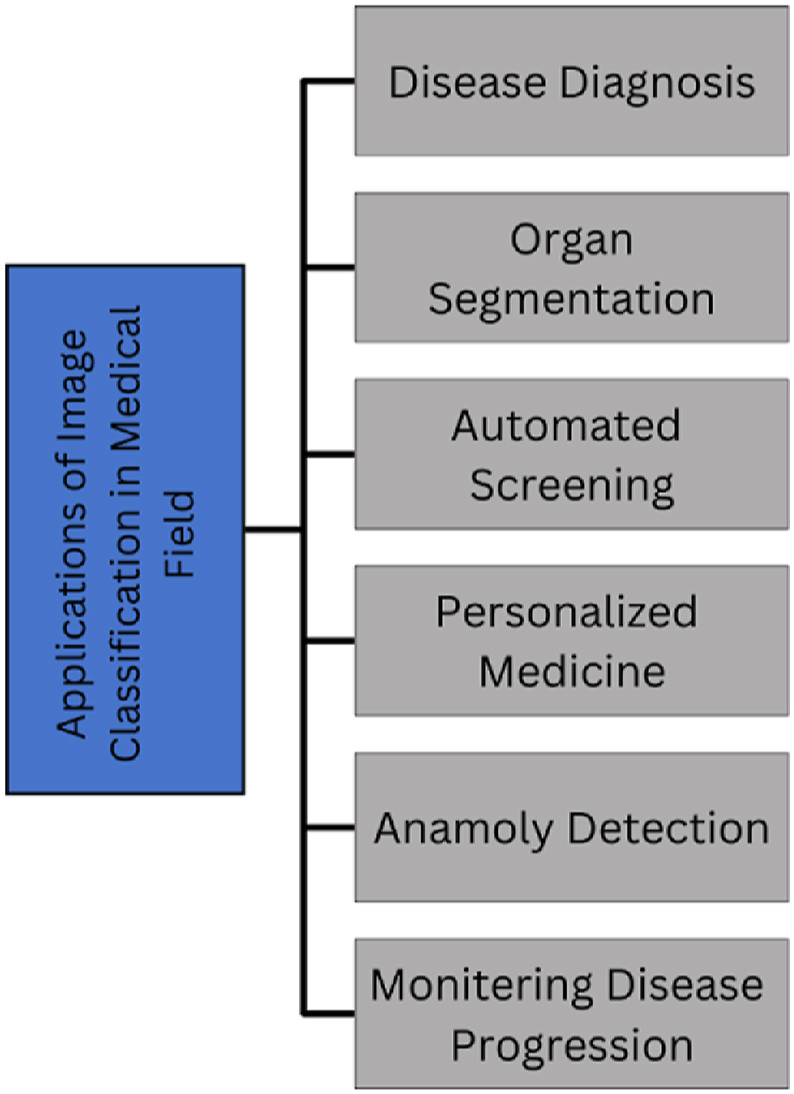
Applications of image classification in medical field.

Nowadays, image classification problems are mostly tackled through deep learning (DL) approaches [1,2]. One of the important roles of DL-based models is learning prominent features specially for solving classification problems. Convolutional neural network (CNN) based variants have been proposed for object detection [3] and image classification [4] tasks. The promising healthcare applications of DL-based methods include automated classification [5,6], disease diagnosis [7], edge devices [8], segmentation [9], and anomaly detection [10]. Fig. 1, graphically demonstrates the significance of image classification for handling different healthcare issues.

**Fig. 2:**
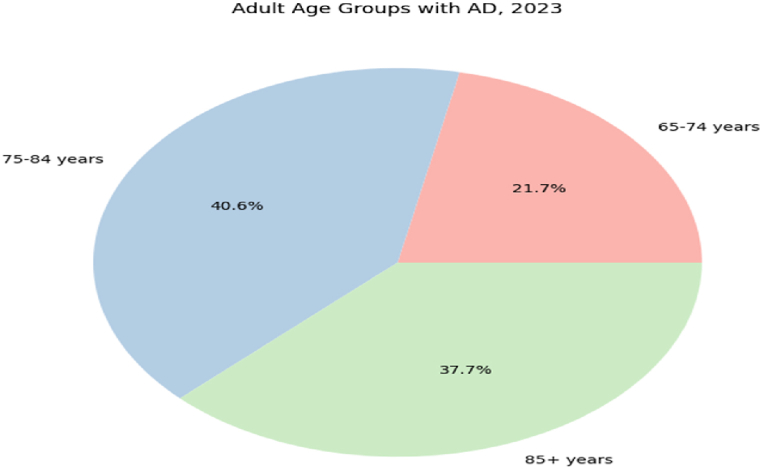
Adults age groups with Alzheimer's disease diagnosis, 2023.

**Fig. 3:**
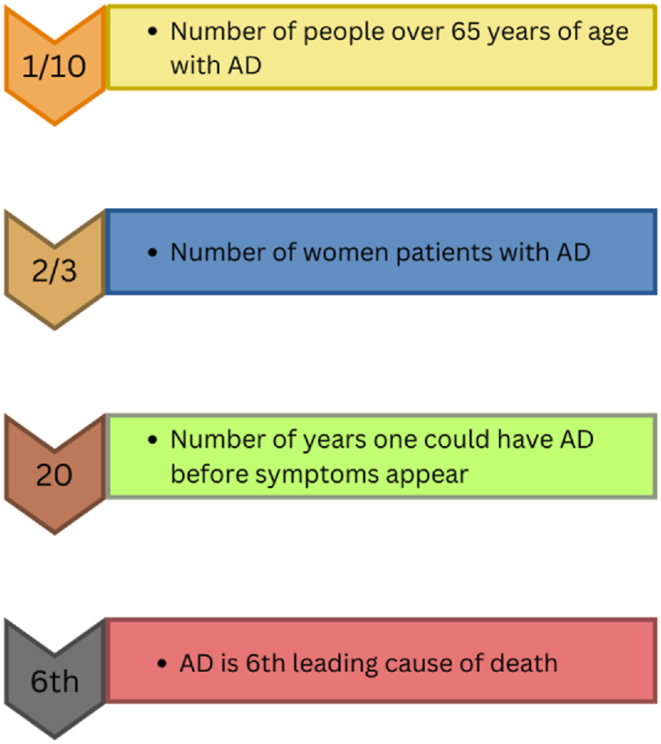
Truths [11] about Alzheimer's disease.

**Fig. 4:**
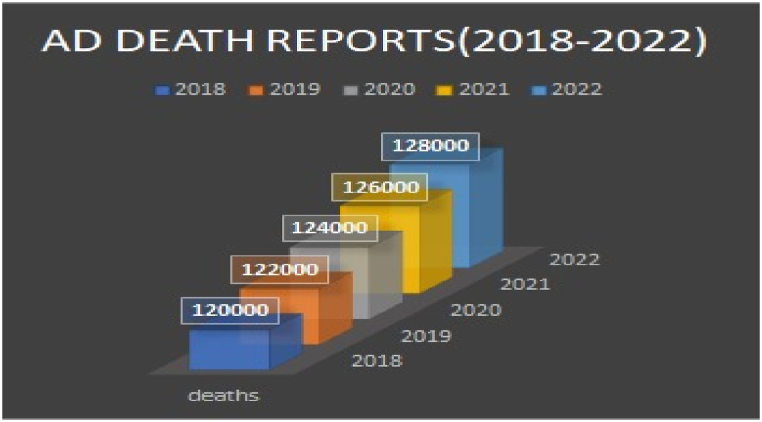
Alzheimer's disease death Reports (2018–2022).

Alzheimer's disease (AD) corresponds to the well-known brain disorder that steadily affects the brain memory of the patients. The mild cognitive impairment (MCI) is referred to as an early stage of AD, though the growth of AD is not certain in patients with MCI. The evolution of AD is witnessed in adults with the age of 65 and above. The proportion of patients affected from AD during the year 2023 are graphically shown in Fig. 2. The hierarchical structure of the brain makes it difficult to detect AD diagnosis by using neuroimaging features. The ground evidences [11] portrayed in Fig. 3 confirm the critical nature and intricacy of the AD diagnosis. The effective treatment for the early AD diagnosis is one of the challenges in the healthcare domain. The number of deaths (62,000) reported in Fig. 4 for five years (2018–2022) were due to the improper AD treatment. The accurate AD detection is required to treat the patients timely and properly.

Researchers have suggested numerous approaches for accomplishing the task of accurate classification of AD diagnosis over the past few years. Different deep learning methods [12] are developed for AD diagnosis classification. It is observed that the existing models are computationally expensive due to their high complexity in the proposed architectures. Despite their complex architectures, the suggested models are incapable of producing interpretable and quality results for accurate classification of AD. The probable challenges of the study include complex CNN-based variants, unexplainable outcomes, accuracy, efficiency, computational cost, adaptability, and robustness.

The goal of this study is to propose a simplified yet effective fractional order-based convolutional neural network (Fr-CNN) model that comprises enhanced attention mechanism, unexploited pooling technique, and novel optimization approach for accurate and efficient AD diagnosis classification. Explainable Artificial Intelligence (XAI)-based Local Interpretable Model-Agnostic Explanation (LIME) approach is incorporated to produce interpretable predictions by the proposed Fr-CNN model. By exploiting LIME, it offers explanation of the decision made by the proposed Fr-CNN model by highlighting the prominent features and portraying the features' contributions.

Recently, fractional order-based stochastic gradient descent (FSGD) [[13], [14], [15]] variant is developed for tackling different problems. Furthermore, a generalized version of fractional-SGD (GFSGD) is suggested in Ref. [16] to further boost the convergence speed and accuracy of recommendations. In this study, we aim to exploit tensorflow framework-based GFSGD variant for image classification to provide accurate and efficient classification through CNN.

The prime objectives of the study are as follows:

• To correctly classify AD at early stages when preventive measures can be effective to overcome further development.
• To propose a simplified yet effective and computationally inexpensive CNN classifier for accurately and efficiently classifying AD diagnosis.
• To increase the classification accuracy by manipulating an effective and reliable pooling technique with enhanced attention mechanism.
• To boost the classification speed by proposing a novel GFSGD optimization technique for AD classification.
• To exploit XAI-based LIME approach for producing interpretable predictions by the proposed Fr-CNN model.
• To increase the robustness of the proposed model as compared to standard methods by discovering the adaptive nature of the optimizer.

### 1.1 Literature review

Janghel et al. [17] suggested a VGG based CNN architecture with multiple classifiers for detecting AD diagnosis at premature stages through MRI analysis. The ADNI database is utilized for AD diagnosis classification task with two class labels of AD and CN (normal control). The recommended model comprises VGG-16 architecture with k-means clustering and SVM classifier for precise classification among AD diagnosis MRIs. Sarraf et al. [18] utilized a multiple pre-trained model for AD diagnosis classification by examining neuroimaging. The scale & shift variant approach is exploited as a feature extraction mechanism. Later, comparing the performance of GoogleNet and LeNet-5 models, it appears that GoogleNet achieves a better accuracy of 98.8 % for AD diagnosis classification. Wu et al. [19], proposed a 3D transfer network in comparison with present 2D transfer network for classifying AD patients with normal individuals. In the suggested technique for feature extraction, 2D networks are incorporated. The extraction output is concatenated and forwarded for categorization purpose. It is depicted that the proposed 3D transfer networks outperform conventional 2D networks with around 10 % accuracy points.

In [20], a 3D Residual RepVGG-based attention network is developed for AD classification. With the incorporation of non-local context spatial attention (NCSA) mechanism within VGG architecture, a satisfactory accuracy is achieved. However, the computational complexity of the ResRepANet model was too high due to more than 7 million architectural parameters. In Ref. [21], authors utilized adaptive synthetic (ADASYN)-based sampling strategy in preprocessing steps for augmentation purposes. Afterwards, four transfer learning (TL)-based pre-trained models such as VGG16, InceptionV3, ResNet50, and Xception are exploited to classify MRIs of AD patients. Moreover, in Ref. [22], the customized architecture is developed by recombining the blocks of CNN and LSTM. Through this ensemble network, authors achieved a remarkable classification accuracy of 98.6 % for AD detection. However, due to the excessive architectural layers in the network, the size of the model was around 165 MB. So, the computational inefficiency was the core limitation in their solution. Furthermore, in Ref. [23], another customized network named as ‘Siamese 4D-AlzNet’ is developed by incorporating the additional convolutional blocks in TL-based models. The Siamese 4D-AlzNet network was extremely complex with more than 15 billion floating point operations (FLOPs) and 470 MB model size. However, the suggested approach reaches a remarkable accuracy of 95.07 %. Similarly, authors of [24] introduce a triplet loss operator driven Siamese CNN model for AD classification. It utilizes triplet loss function to represent the MRIs in embedding forms. This approach displays satisfactory performance by achieving 91.83 % accuracy on ADNI dataset.

Al-khuzaie et al. [25] suggested a novel ‘AlzNet’ CNN-based model for classification among AD and CN. The architecture comprises 5 conv layers and max-pooling layers with ReLU as the activation function. The OASIS dataset is exploited and the model is trained on 15,200 images. The best accuracy of 99 % is attained by the proposed AlzNet model for binary classification task. Safi et al. [26], proposed a unique approach for AD diagnosis through EEG signal analysis. Various signal processing strategies like empirical mode decomposition (EMD) and discrete wavelet transform (DWT) are incorporated with multiple classifiers such as SVM and KNN for accurate and efficient AD detection. The findings of the study include an improved accuracy in terms of AD detection. Lu et al. [27] present a study of comparison between MobileNet and VGG16 architectures for AD classification. VGG16 is considered as a state-of-the-art (SOTA) architecture and baseline model for the study. With MobileNet architecture, it achieves a better accuracy of 94 % as compared to the baseline model for accurate classification of AD through neuroimaging analysis.

Antony et al. [28], suggested VGG family architectures for AD diagnosis classification. In preprocessing steps, data augmentation technique and skull stripped mechanism are incorporated for 780 MRIs of ADNI database. Due to the VGGs' complex and computationally expensive models, better results are not generated with the suggested approach. The best accuracies of 81 % and 84 % are achieved by VGG-16 and VGG-19 models. Mujahid et al. [29] introduced an ensemble learning strategy for the classification of AD diagnosis. Two heavy CNN-based architectures like VGG16 and EffecientNet-B2 are ensembled for premature stage AD detection. OASIS dataset is utilized in the study. In order to overcome the imbalance distribution challenge within the dataset, an adaptive oversampling approach is exploited. The accuracy of 97.35 % is attained by the proposed technique.

Duc et al. [30] present a novel approach for automatic detection of AD using MRIs through a 3D-based deep learning technique. Multiple feature extraction & optimization approaches like SVM-RFE and selection operator are exploited for enhanced feature extraction through improved information focus. The accuracy of 86.12 % is achieved by the suggested 3D-based deep learning method. Basheera et al. [31] proposed a novel custom CNN model for AD classification. The residual and inception blocks are incorporated within CNN architectures for enhanced feature extraction and better dimensionality reduction. The enhanced independent component analysis (EICA) technique is exploited for segmentation mask on MRI images for better visualization. The suggested approach is executed for both multi-class and binary classification of AD. The accuracy of 86.7 % is achieved by the proposed technique. Ben Ahmed et al. [32] introduced a hippocampal visual features (HVF) mechanism within the classifier model for enhanced prominent features extraction from an input MRI image. By exploiting HVF, an improved accuracy is reached by the model while distinguishing between Alzheimer's disease and mild cognitive impairment patients. The accuracy of 87 % is attained by the proposed study. Similarly, Venugopalan et al. [33] exploited denoising auto-encoders for prominent feature extraction within neuroimaging data. Afterwards, the study utilizes multiple classifiers such as KNN, SVM, and random forest during critical analysis of the study. The best accuracy of 88 % is achieved with the SVM classifier.

Feng et al. [34] recommended a 3D-CNN model for AD detection. A CNN architecture's hidden layers are incorporated for feature extraction and dimensionality reduction. A separate SVM classifier is utilized for the classification of AD in the study. With the suggested method, an accuracy of 92.1 % is achieved. Maqsood et al. [35] utilize transfer learning based AlexNet pretrained model for AD diagnosis classification. The model is trained on segmented images and evaluated on unsegmented MRI scans. The model is executed for both binary and multi-class classification in the study. The accuracies achieved by the model are 92.8 % and 89.6 % for multi-class and binary classification, respectively. Similarly, Savas et al. [36], explored pre-trained CNN architectures like ResNet-50, DenseNet, XceptionNet, and EfficientNet for AD classification. After comprehensive training, it is depicted that EfficientNetB3 model outperforms other models in terms of accuracy by achieving best accuracy of 92.98 % for AD classification.

Choi et al. [37] proposed an ensemble learning concept for AD classification. Multiple deep CNN architectures ensembled with novel ensemble generalization loss (EGL) are exploited for weight optimization. The classification accuracy achieved by the suggested ensemble model is 93.84 %. Jain et al. [38] presented a study on VGG-16 architecture for AD detection and classification. Feature extraction is performed through VGG-16 hidden layers and dense layers are used for classification of AD and MCI among different MRIs. An accuracy of 95.7 % is achieved in the proposed study. Peng et al. [39] introduced a regularized kernel learning-based approach for AD classification. Kernal learning is incorporated as a feature extraction mechanism in the architecture by extracting most prominent features or hidden patterns within MRIs. The best accuracy of 96.1 % is achieved by the proposed kernel-based approach. Hon et al. [40] exploited pre-trained models such as VGG-16 and Inception V4 for AD diagnosis classification. The results with VGG-16 model were not satisfying but with InceptionV4, better accuracy of 96.25 % was achieved for AD classification. Similarly, Shanmugam et al. [41] and Ebrahimi et al. [42] presented a study on GoogleNet and ResNet-18 pretrained model for AD classification. The accuracy of 96.3 % and 96.8 % are attained for the multi-class classification task by exploiting GoogleNet and ResNet-18 architectures.

In [43], a transformer driven pre-trained DeiT model is exploited for AD classification. The robustness of the DeiT model was verified through the addition of noise by fluctuating number of slices. The suggested approach reaches an accuracy of 76 % for classifying MRIs of AD patients. Moreover, in Ref. [44], an ensemble learning approach is explored for the classification of AD and prediction about MCI conversion. The study presents a mixture of regression-classification strategy, in which the suggested ensemble architecture is utilized to classify the MRIs with AD diagnosis and separately predicts about MCI conversion. However, due to the complexity of the architecture, the size of the suggested network was around 60 MB with 15.2 million architectural parameters, which shows that the suggested approach was not reliable and cost-effective in terms of practical deployment on resource-friendly devices.

Beheshti et al. [45], proposed a novel genetic algorithm for AD diagnosis classification. By exploiting the genetic approach for AD classification, an improved accuracy of 97.4 % is reached by the suggested model as compared to baseline models. Raju et al. [46] introduced an innovative 3D-CNN model for AD classification. The hidden layers of CNN are utilized for feature extraction and SVM-based classifier is incorporated for classification. The accuracy of 97.7 % is achieved by the proposed custom 3D-CNN model. Raza et al. [47] proposed a transfer learning approach for AD classification through neuroimaging analysis. Gray Matter (GM) based segmentation technique is utilized for performing image segmentation on MRIs of ADNI database. The accuracy of 97.84 % is achieved by the recommended study after 50 iterations. Illakiya et al. [48] suggested an adaptive hybrid attention based ‘AHANet’ model for AD classification. The attention mechanism is incorporated for enhanced information focus. The classification accuracy achieved by the proposed model is around 98.5 % for AD classification. Similarly, Bhuvaneswari et al. [49] exploited principal component analysis (PCA) for better extraction of most prominent features within MRIs. By utilizing PCA, the proposed model shows enhanced performance by accurately classifying AD with the classification accuracy of 98.7 %.

Recently, various segmentation driven approaches are introduced to identify the certain regions of MRI such as cortical, hippocampal, and ventricle regions. These regions are mainly identified through segmentation techniques to gain valuable insights about the structural variations that happen in brain due to the progression of AD. In Ref. [50], authors segmented the hippocampal regions of human brain to understand the progression of AD. Similarly, in Ref. [51], a hybrid deep architecture is developed to initially segment the hippocampal region from input MRIs and then predict about the AD chances on the basis of the segmented MRIs. Furthermore, in Ref. [52], authors designed a fuzzy C-means clustering strategy to segment the brain tissues in human brain, which provide valuable information about the structural atrophy linked with Alzheimer's disease. Furthermore, machine learning models have been constructed to categorize other tumor-based diseases, such as skin cancer [53], lung cancer [54], colorectal cancer [55], and cataract and glaucoma cancer detection [56].

Fractional calculus-based [57] solutions have gained significant attention due to their strong mathematical foundations. Fractional calculus concepts have been exploited for solving problems in various domains including disease modeling [[58], [59], [60]], corruption dynamics [61], power systems [62], medical field [63], epidemic systems [64], control systems [65], heat transfer analysis [66], boundary problems [67,68], nonlinear systems [69], and biological systems [70]. To achieve better convergence speed and estimated accuracy, researchers have introduced fractional gradient-based optimizers for solving different problems, such as communication [71], parameter estimation [72,73], output error model identification [74], neural networks [[75], [76], [77]], fuzzy functions [78], Laplace transform [79], and recommender systems [[13], [14], [15]]. Recently, fractional order-based implementations of standard optimizers like FSGD and FADAM for solving deep neural network problems are provided in Ref. [80] using pytorch framework. Moreover, a generalized fractional variant of standard stochastic gradient decent (GFSGD) is proposed in Ref. [16] to provide a computationally inexpensive solution by offering improved convergence speed and accuracy. The proposed GFSGD is further explored for efficient matrix factorization in recommender systems [81]. The improved performance of GFSGD in terms of speed and accuracy motivates us to investigate the potential of fractional gradient based optimizer for AD diagnosis classification.

### 1.2 Our contributions

The key contributions of the proposed study are as follows:

• A simplified CNN architecture for accurate and efficient classification of AD is proposed. The proposed approach is novel in terms of architecutre with effective pooling technique and improved attention mechanism for diverse feature extraction.
• A tensorflow framework-based generalized version of fractional SGD (GFSGD) is developed to enhance the classification speed.
• Explainable AI powered CNN classifier is designed for interpretable AD diagnosis classification.
• The proposed Fr-CNN model outclasses complex benchmark models in terms of evaluation metrics on standard ADNI dataset.

### 1.3 Paper organization

The contents of the paper are organized in the following manner: The architecture of the proposed Fr-CNN model is described in Section 2. Afterwards, the critical analysis of the results and comparison with benchmark models is performed in Section 3. The findings of the study are elaborated in Section 4. Finally, the conclusions are presnted in Section 5 along with potential future directions.

## 2 Architecture of the proposed model

**Fig. 5:**
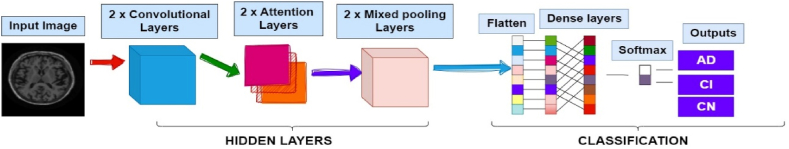
General architecture diagram of proposed Fr-CNN.

The capabilities of CNN are utilized for accomplishing the task of accurately classifying AD diagnosis by analyzing given MRIs. The attention mechanism is incorporated within the proposed Fr-CNN architecture to enhance the performance of the model. The attention module comprises a single convolutional filter with the size of 1∗1 for an input tensor, using a sigmoid activation function. The outcome of this operation is multiplied with the original input tensor for feature mapping process. The multiplied result with dimension vector is the final output of the attention mechanism, which is forwarded to the layer ahead within the architecture. The general architecturaldiagram of the proposed Fr-CNN model is presented is Fig. 5. The architecture comprises 2 convolutional, attention, and mixed pooling layers. First conv layer with 200∗3∗3 and second with 100∗3∗3 filter size are used with regular CNN computations. An effective mixed pooling technique with 2∗2 pool size is incorporated in the architecture for prominent feature extraction and dimensionality reduction task. The model utilizes ReLU as the activation function. The attention module is placed between convolutional and pooling layer in architecture for better information focus. A sandwich-like structure is proposed in the context of these hidden layers. Furthermore, the proposed architecture incorporates 3 dense layers, which are influential in learning the complex hidden patterns within human MRIs from the training image dataset and resulting in a significant performance by the proposed Fr-CNN model with regards to predictions on unseen images. Softmax activation function is incorporated in the last fully-connected layer for effective probability-based classification. The overall architectural parameters of the proposed Fr-CNN model are listed in Table 1.

**Table 1:** Architecture parameters of proposed Fr-CNN model.

Layer (type)	Output Shape	Parameters
Conv2D	(None,126,126,200)	5600
Attention	(None,126,126,200)	201
MixedPooling	(None,63,63,400)	0
Conv2D-1	(None,61,61,100)	360100
Attention-1	(None,61,61,100)	101
MixedPooling-1	(None,30,30,200)	0
Flatten	(None,180000)	0
Dense	(None,100)	18000100
Dense-1	(None,50)	5050
Dense-2	(None,3)	153
Total params: 18371305 (70.08 MB) Trainable params: 18371305 (70.08 MB) Non-trainable params: 0 (0.00 Byte)		

### 2.1 Unexploited pooling method

The utilized mixed pooling is achieved by average and max pooling techniques. The diverse nature of customized mixed pooling method offers essential benefits in image classification tasks.

• **Diverse information extraction:** Combination of multiple pooling methods result in extracting most prominent information with smooth representation of the desired region.
• **Overcome overfitting:** Mixture of operations assists as a regularization technique within neural architecture, which avoids the proposed model to be overfitted.

### 2.2 Attention module

Transformer architectures are popular for their self-attention mechanisms. In this study, a spatial attention mechanism is implemented by using a convolution followed by a sigmoid attention function. Given an input feature map **X** of shape (H, W, C), where H and W are the height and width, and C is the number of channels, the attention mechanism **A** is formulated as A=σ(W∗X+b), where W represents a convolutional kernel of the shape (1,1,C,1), **b** is the bias term, σ denotes the sigmoid activation function, and ∗ is the convolutional operator. The resulting attention map is **A** of shape (H, W, 1), where each individual element $a_{ij}\in \left[0,1\right]$. The attention map **A** is multiplied elementwise with the original input feature map **X** resulting in the attended feature map $X^{\prime }=X⊙A$. This way the important regions are emphasized ($a_{ij}$ is closer to 1) and the less important regions are suppressed ($a_{ij}$ is closer to 0). This attention mechanism is incorporated within the simplified CNN to boost the performance of the proposed model.

• **Enhanced information focus:** Attention layers allow the model to focus on the prominent features region within the input image.
• **Increased robustness and adaptability:** It permits the model to disregard the unrelated share of the input image, which may enhance model's robustness. Besides, attention weights aid a degree of adaptability, making it easier to distinguish which region of the input the model deems significant for a given prediction.

### 2.3 Explainable AI

**Fig. 6:**
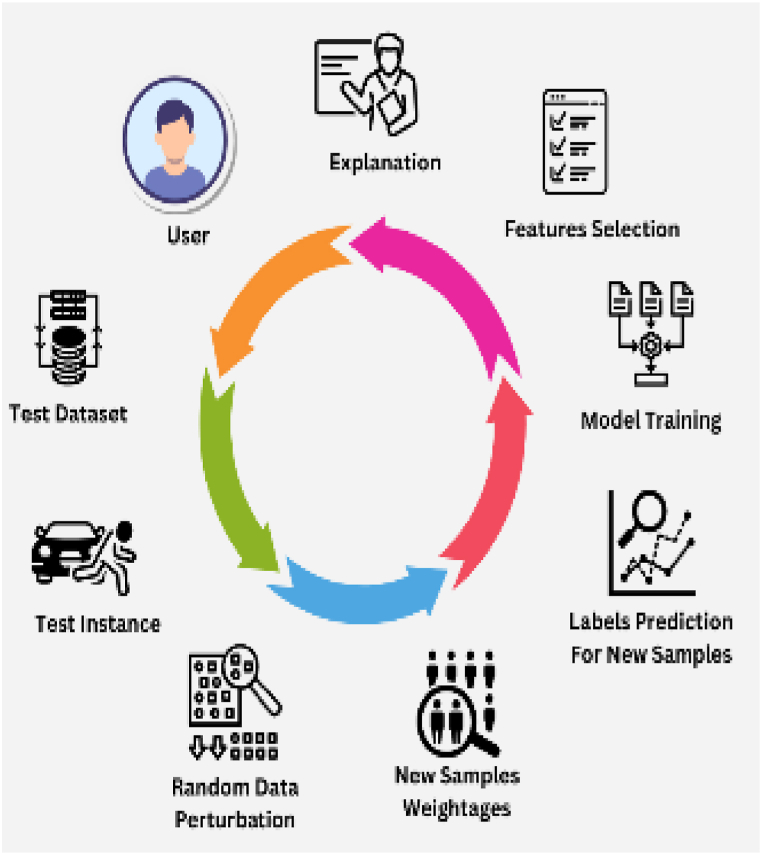
Graphical Abstract of LIME

Explainable AI (XAI) driven Local Interpretable Model-Agnostic Explanation (LIME) based method is exploited in the study. LIME provides transparency to the model, which improves the model's analytical proficiencies. LIME enlightens the performance of the model in terms of its decision-making procedure. It emphasizes on local actions of the model to produce human-understandable clarifications for any precise estimate by highlighting the section with most prominent features and their contributions behind the accurate predictions. LIME chooses the instance for which the explanation is needed; in our case, it is the desired image from test dataset. Afterwards, it generates a perturbed dataset by changing the feature values of the given instance, but the truth label remains same for each perturbed sample as that of original instance label. Later, the perturbed dataset and image of the original instance is fed to the proposed model for prediction. The previous labels of perturbed dataset are then updated with the labels predicted from the proposed model. Furthermore, LIME incorporates a linear model like decision trees. The updated perturbed dataset except the original instance image is used to train the local model. After comprehensive training process, original instance is fed to the local model for predictions. LIME investigates the local behavior of the model and coefficient parameters with the proposed model to figure out the prominent features region within the input image. LIME graphically represents the positive feature contributions inside the image. By exploiting LIME-based XAI, the proposed model generates interpretable and explainable predictions on any test image. The graphical working process of LIME is shown in Fig. 6.

### 2.4 Generalized Fractional Stochastic Gradient Descent (GFSGD)

This section delves into the Generalized Fractional Stochastic Gradient Descent (GFSGD) optimization algorithm. A rigorous mathematical derivation of the update rule for GFSGD is presented, along with TensorFlow-like pseudocode tailored for practical implementation.

#### 2.4.1 Mathematical foundations behind the GFSGD method

We know that the update rule to find the minimum of a function g(⋅) using the well-known gradient descent methods is as follows:

(1) $$x_{e+1}=x_{e}-\rho \nabla g\left(x_{e}\right)$$

where.

• $x_{e}$ and $x_{e+1}$ represent the parameters of the function g(⋅) at the current epoch e and the next epoch e+1, respectively.
• ρ is the learning rate, where ρ>0 determines the step size at each iteration.
• $\nabla g\left(x_{e}\right)$ is the gradient of the function g(⋅) with respect to x at epoch e, evaluated at the current parameter value $x_{e}.:$:

The main intuition behind the GFSGD is to replace the conventional derivative with a fractional derivative with their characteristic memory. Fractional calculus extends the gradient to a non-integer domain, allowing for the integration of past system states into current evaluations. With substituted fractional gradient into (1), the update rule becomes

(2) $$x_{e+1}=x_{e}-\rho \nabla^{\alpha }g\left(x_{e}\right)$$

where $\nabla^{\alpha }g\left(x_{e}\right)$ is the gradient of the function with fractional order α. We use the Caputo definition of the fractional derivative for the derivation of the update rule for the GFSGD method, which is given as

(3) Dxαacg(x)=1Γ(n−α)∫ax(x−τ)n−α−1g(n)(τ)dτ

where n−1<α<n, $n\in N_{+}$ and a represents the lower bound of the integral and x is the current position for the continuous function g(⋅). The expression Γ(n−α) in (3) represents the notation of Gamma function. The integral illustration of Gamma function Γ(n−α) can be generally expressed as: $\Gamma \left(n-\alpha \right)=\int_{0}^{\infty }s^{\left(n-\alpha \right)-1}e^{-s}\text{ds},\text{for}\;\text{Re}\left(n-\alpha \right)>0$. For the discrete case, (3) becomes:

(4) Dacxeαg(xe)=1Γ(n−α)∫axe(xe−τ)n−α−1g(n)(τ)dτ

Taylor series expansion of the function g(⋅) at $x_{e}$ can be used to rewrite the equation as equation (3) in Ref. [82].

(5) Dacxeαg(xe)=1Γ(n−α)∫axe(xe−τ)n−α−1g(n)(τ)dτ=1Γ(n−α)∫axe∑j=0+∞(−1)jg(j+n)(xe)Γ(j+1)(xe−τ)n+j−α−1dτ(Taylorexpansionatxe)=1Γ(n−α)∑j=0+∞(−1)jg(j+n)(xe)Γ(j+1)∫axe(xe−τ)n+j−α−1dτ(Interchanging∑and∫)=1Γ(n−α)∑j=0+∞(−1)jg(j+n)(xe)Γ(j+1)(xe−a)n+j−αn+j−α(Solvingtheintegral)=∑j=n+∞(−1)jg(j)(xe)(n−α)Γ(j−n+1)(j−α)(xe−a)j−α(Reindexingthe∑fromj=0toj=n)=∑j=n+∞(α−nj−n)g(j)(xe)Γ(j−α+1)(xe−a)j−α(Simplifyingusingbinomialcoefficient)

where n−1<α<n, $n\in N_{+}\text{,}$ and the binomial coefficient $\left(\begin{array}{l} a\\ b \end{array}\right)=\frac{\Gamma \left(a+1\right)}{\Gamma \left(b+1\right)\Gamma \left(a-b+1\right)}$. It is intuitive to use the short memory principle (SMP) by replacing the lower bound a with $x_{e-E}$ where E is the fixed memory length [83,84]. SMP is employed to reduce the computational burden and emphasize recent information. The adoption of fixed memory E=1, as supported by findings in Ref. [85], involves using only the immediate previous change in the function. This approach further aids in preventing overshoot and further curtails computational demands. The calculations in equation (5) can be repeated with a different lower bound $x_{e-1}$, the resultant takes the form:

(6) Dxe−1cxeαg(xe)=∑j=n∞(α−nj−n)g(j)(xe)Γ(j−α+1)(xe−xe−1)j−α.

For an optimization problem, if the first derivative $g^{\left(1\right)}\left(x_{e}\right)$ becomes zero, we have effectively found the solution, so we can truncate the higher order terms in the summation. By effectively using only the first order term (j=1), we get

(7) Dxe−1cxeαg(xe)=g(1)(xe)Γ(2−α)(xe−xe−1)1−α

where 0<α<1. To help prevent a complex case, we can add an absolute for the difference in the current and previous parameters such that $x_{e}-x_{e-1}>0$ is always true. By introducing this, we effectively extend the range of α to 0<α<2. We can also introduce a small term $\epsilon =10^{-8}$ to the power to prevent 0 in the denominator for the case where $x_{e}=x_{e-1}$.

(8) Dxe−1cxeαg(xe)=g(1)(xe)Γ(2−α)(|xe−xe−1|+ϵ)1−α.

By replacing (8) in (2), we essentially get the update rule of GFSGD

(9) $$x_{e+1}=x_{e}+\frac{\rho g^{\left(1\right)}\left(x\right)}{\Gamma \left(2-\alpha \right)}{\left(\left|x_{e}-x_{e-1}\right|+\epsilon \right)}^{1-\alpha }$$

GFSGD update rule is a merge between Theorem 1 and Theorem 2 from Ref. [67]. It utilizes both the fixed memory step along with higher order truncation. You can also observe that for α=1, equation (9) becomes equivalent to the standard gradient descent method.

(10) $$x_{e+1}=x_{e}+\rho g^{\left(1\right)}\left(x\right)$$

#### 2.4.2 Pseudocode for GFSGD

For TensorFlow implementation of GFSGD, we mainly modify *update_step* of the standard SGD in a Tensorflow code-like pseudocode, as follows:

**Figure:**
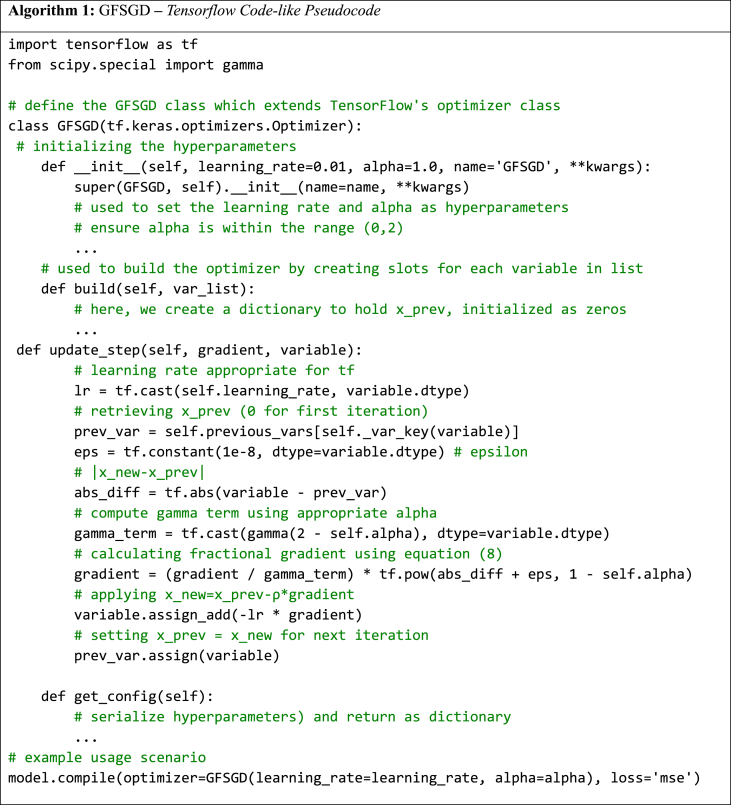

## 3 Results and discussion

This section comprises database description, critical analysis of the proposed Fr-CNN model in terms of results, and performance comparison with benchmark models.

### 3.1 Dataset description

**Fig. 7:**
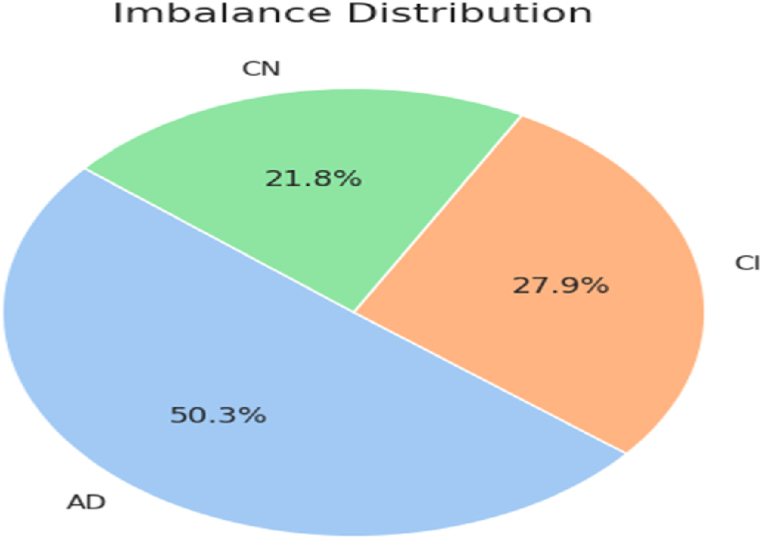
Imbalanced class distribution in ADNI dataset.

**Fig. 8:**
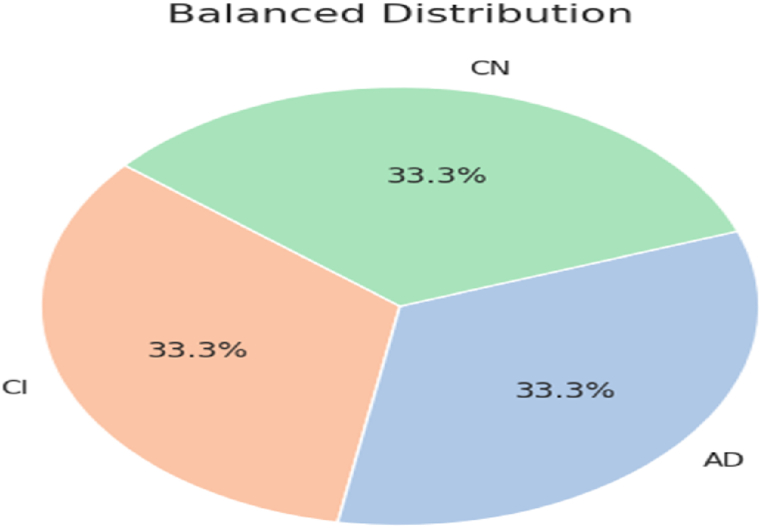
Balanced Distribution of classes for AD classification task.

**Fig. 9:**
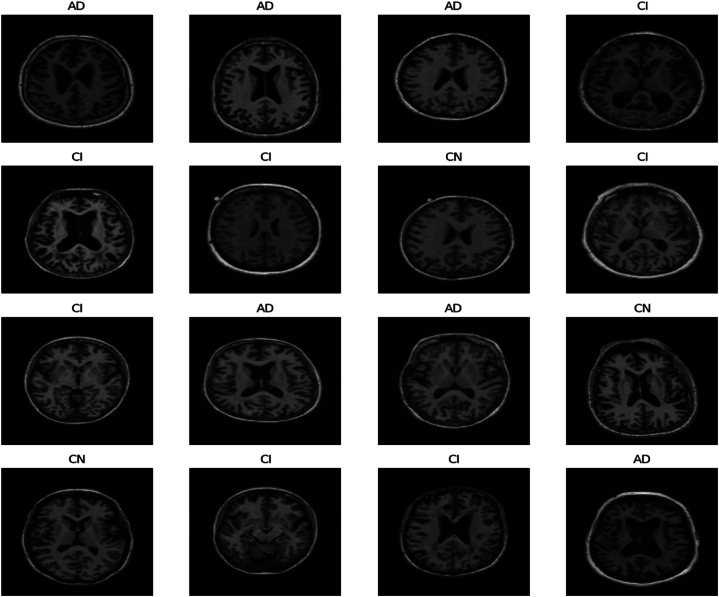
Sample Neuroimages from standard ADNI database.

The study utilizes ADNI [86] database, best available for Alzheimer's disease diagnosis tasks. The database comprises 3 classes: AD (patients with Alzheimer's disease), CI (patients with mild cognitive impairment), and CN (common normal individuals). ADNI database contains 5154 MRIs in total. Fig. 7 shows the distribution of each class label with respect to MRIs. It is evident that initially, the database has imbalanced class distribution, which is not preferred for classification tasks. In order to make the uniform number of MRIs for all class labels, the data augmentation techniques (horizontal and vertical random flip) are exploited to oversample the MRIs of various classes. Afterwards, 3002 samples for each class are considered for further progress in the study. Subsequently, pixel normalization is applied to produce pixel values in the range [0,1]. The balanced distribution of each class with respect to MRI scan images is represented in Fig. 8. Sample MRI scans of each class in ADNI dataset are given in Fig. 9, to offer the valuable insights about the dataset in terms of distinct visual attributes of each class and quality, or resolution of MRI scans. In Fig. 9**,** the MRIs of each AD diagnostics class is presented to notify the difficult characteristics/features that the model needs to learn in order to accurately classify different stages of AD. The overlapping characteristics of MRI scans for each class label shown in Fig. 9 underscore the enriching capabilities of the proposed approach in accurately and efficiently classifying the progression of Alzheimer's disease. Overall, the ADNI database emerges as a best available dataset for any research work in the field of AD.

### 3.2 Evaluation measures

The performance of the proposed Fr-CNN model is validated through diverse evaluation measures given in Table 2, which are computed through the formulation of true positives (Tr_Ps), true negatives (Tr_Ng), false positives (F_Ps), and false negatives (F_Ns).

**Table-2:** Evaluation metrics.

**Accuracy (A)**	$\frac{Tr\_Ps+Tr\_Ng}{Tr\_Ps+Tr\_Ng+F\_Ng+F\_Ps}$
**Precision (P)**	$\frac{Tr\_ps}{Tr\_Ps+F\_Ps}$
**Recall (R)**	$\frac{Tr\_Ps}{Tr\_Ps+F\_Ng}$
**F1-Score (F_S)**	$2\times \frac{P\times R}{P+R}$

### 3.3 Critical analysis of the proposed model

The proposed Fr-CNN model is trained on ADNI dataset with 85 % and 15 % split for training and testing sets, respectively. Additionally, 15 % of the training is reserved as validation set. Both splits are stratified to maintain consistent class distribution across the three sets. By exploiting improved attention mechanism and more effective mixed pooling technique in the architecture, enhanced feature extraction task is accomplished, which leads to a substantial performance by the proposed model. In order to make the model computationally inexpensive, fast optimizer GFSGD is incorporated in the study with different alpha variations, to analyze the convergence speed and trends of the proposed model. Initially, the Fr-CNN model is executed with standard stochastic gradient descent (SGD) with the learning rate of 0.001, and the model is compiled for 35 iterations. It is observed that the convergence speed is very slow, the max-val accuracy of 0.68 %, and min-val loss of 0.78 is computed during the execution. Afterwards, the study incorporated Generalized Fractional Stochastic Gradient Descent (GFSGD) optimizer for the better convergence speed and improved generalization by the proposed model. Alpha parameter of GFSGD ranges from 0 to 2; it is observed that with low alpha rates, the convergence speed is not impressive and GFSGD performs best between the alpha rates of 0.8–1.5. Initially, the Fr-CNN model is implemented with GFSGD having alpha of 0.8 with learning rate of 0.001. With alpha of 0.8, the model achieves best accuracy of 0.52 % and loss of 1.05 after 35 epochs. It is depicted that the model with GFSGD having alpha rate of 0.8, the results are even worse as compared to standard SGD with same learning rate and epochs. Later, the proposed model is executed with GFSGD having alpha rates of 0.9 and 1.0. The Fr-CNN model achieves maximum accuracy of 0.58 % and loss of 0.91 after 35 epochs with an alpha rate of 0.9. Then, the accuracy of 0.80 % and loss of 0.53 is attained with alpha rate of 1.0, which are the best results of the study until now. It is observed that as the alpha rates are gradually increasing, the proposed model with GFSGD improved its convergence speed and attained good results by reaching local minima quickly, resulting in computationally effective solution. Seeing the improved results, the proposed model is further executed with GFSGD having alpha rates of 1.1 and 1.2. After same 35 epochs and learning rate of 0.001, the model achieves an accuracy of 0.95 % for both 1.1 and 1.2 alpha rates. The minimum loss reached by the model is 0.18 and 0.15 with alpha rates of 1.1 and 1.2, respectively. It is noted that the convergence speed is extremely boosted as the alpha rate reaches the value of 1.0 in GFSGD. In order to make the proposed Fr-CNN model computationally inexpensive with improved results, the study further tested the higher alpha rates for the GFSGD.

**Fig. 10:**
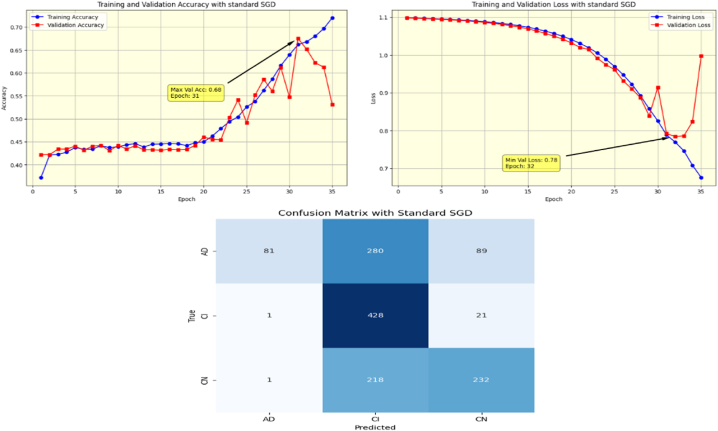
Accuracy and Loss graphs, and Confusion matrix of the proposed Fr-CNN model with SGD.

**Fig. 11:**
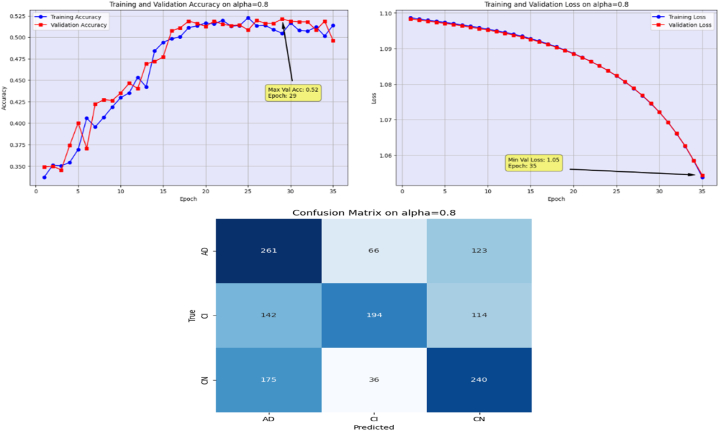
Accuracy and Loss graphs, and Confusion matrix of the proposed Fr-CNN model with GFSGD (alpha = 0.8).

**Fig. 12:**
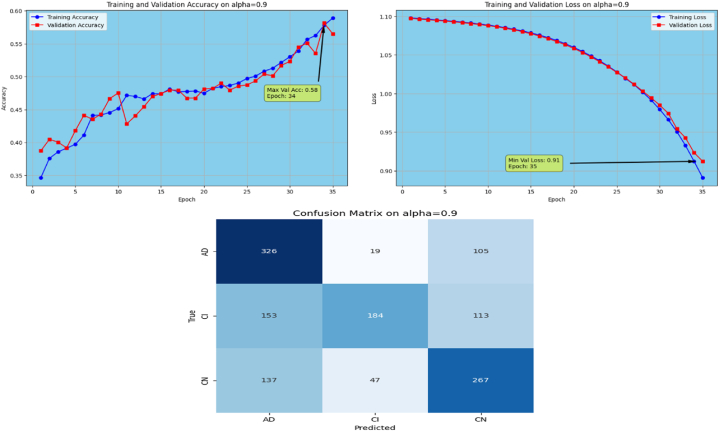
Accuracy and Loss graphs, and Confusion matrix of the proposed Fr-CNN model with GFSGD (alpha = 0.9).

**Fig. 13:**
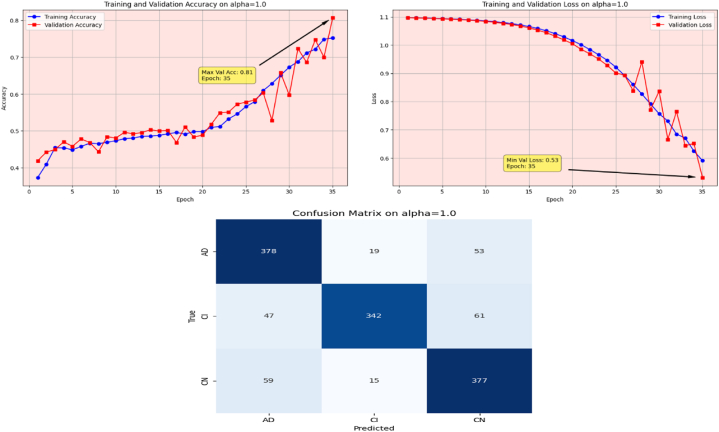
Accuracy and Loss graphs, and Confusion matrix of the proposed Fr-CNN model with GFSGD (alpha = 1.0).

**Fig. 14:**
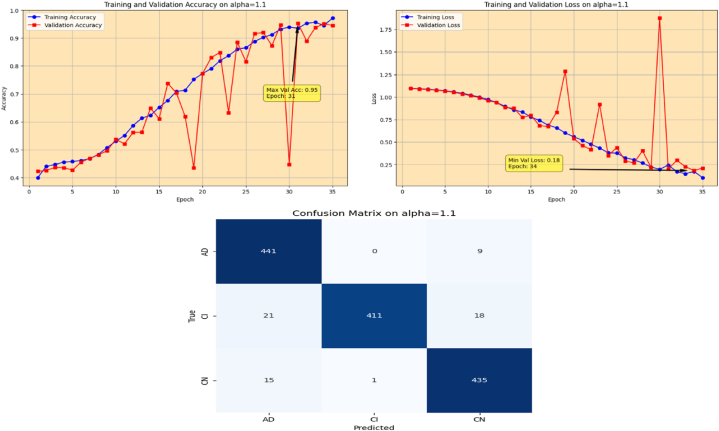
Accuracy and Loss graphs, and Confusion matrix of the proposed Fr-CNN model with GFSGD (alpha = 1.1).

**Fig. 15:**
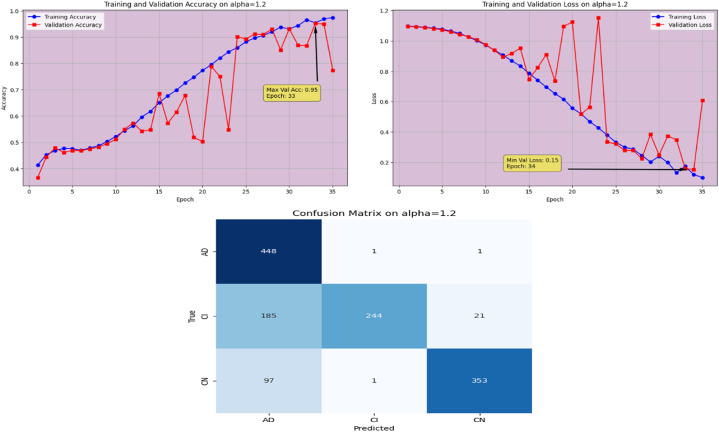
Accuracy and Loss graphs, and Confusion matrix of the proposed Fr-CNN model with GFSGD (alpha = 1.2).

**Fig. 16:**
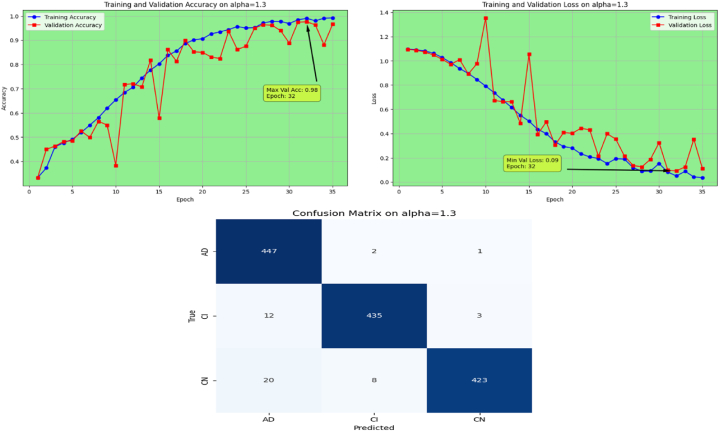
Accuracy and Loss graphs, and Confusion matrix of the proposed Fr-CNN model with GFSGD (alpha = 1.3).

**Fig. 17:**
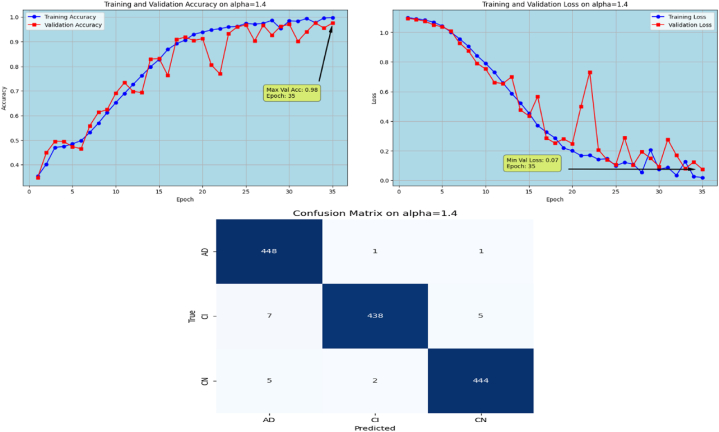
Accuracy, Loss graphs and Confusion matrix of proposed Fr-CNN model with GFSGD (alpha = 1.4).

**Fig. 18:**
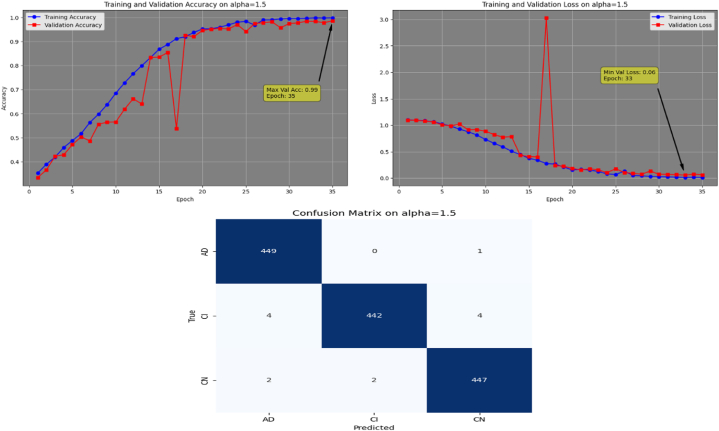
Accuracy and Loss graphs, and Confusion matrix of the proposed Fr-CNN model with GFSGD (alpha = 1.5).

**Fig. 19:**
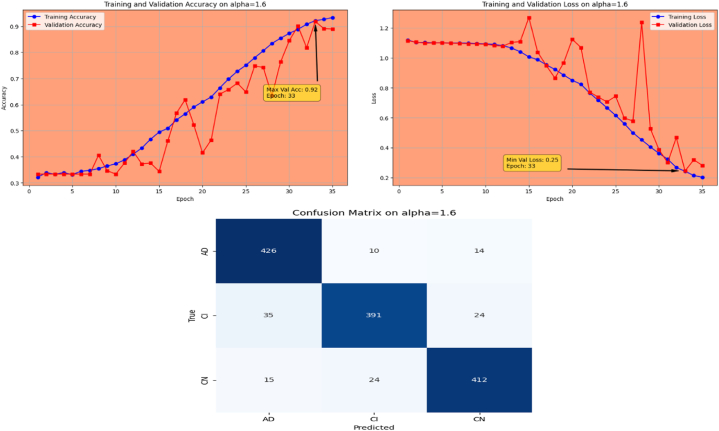
Accuracy and Loss graphs, and Confusion matrix of the proposed Fr-CNN model with GFSGD (alpha = 1.6).

Afterwards, the model achieves max-val accuracy of 0.98 % and min-val loss of 0.09 after only 32 epochs with GFSGD having alpha rate of 1.3. Later, the proposed model is executed with an alpha rate of 1.4 for GFSGD. It attained a max-val accuracy of 0.98 % and min-val loss of 0.07 after 35 iterations. After observing the improved convergence speed by the alpha rates of 1.3 and 1.4, it further motivated to go in depth of GFSGD with higher alpha rates, to achieve the optimal results. Thus, the proposed Fr-CNN model achieved an optimal accuracy of 0.99 % and loss value of 0.06 after 35 epochs when executed with GFSGD having alpha value of 1.5 and learning rate of 0.001. Later, it is noted that as the model executes with alpha value of 1.6, its convergence speed gradually decreases, which results in bad results as compared to alpha values of 1.5 and 1.4. The proposed model attained an accuracy and loss of 0.92 % and 0.25, respectively. It is analyzed that with alpha values of above 1.5, the GFSGD convergence speed reduces, producing a bad impact on the performance of the model. After comprehensive testing of the Fr-CNN model, with different alpha values and learning rate variations, it seems that the proposed best-fit model is with alpha value of 1.5 having learning rate of 0.001, for the given task. With variation in alpha values, the accuracy and loss trends seem to be changing for each case as with its convergence speed. With the change in variations, the model's predictive capabilities also seem to be modified, which can be analyzed by Fig. 10, Fig. 11, Fig. 12, Fig. 13, Fig. 14, Fig. 15, Fig. 16, Fig. 17, Fig. 18, Fig. 19, demonstrating the trends of accuracy, loss graphs, and confusion matrix of the proposed Fr-CNN model for each alpha variation. The evaluation metrics for the classification of AD for each variation with respect to standard SGD and alpha variations in GFSGD are enlisted in Table 3. After analyzing the graph trends, evaluation metrics, and confusion matrix, it is concluded that with the alpha value of 1.5 in GFSGD, the performance and convergence speed of the proposed Fr-CNN model outclasses the standard SGD and produces exceptional results in the context of AD classification.

**Table 3:** Comparison of the Proposed Model with standard SGD and variations in alpha rates for GFSGD in terms of Evaluation Metrics.

Optimizer Variations	Class Labels	Precision	Recall	F1_score	Accuracy (%)
Standard SGD	AD	0.98	0.18	0.30	0.55
	CI	0.46	0.95	0.62	
	CN	0.68	0.51	0.59	
GFSGD (alpha = 0.8)	AD	0.45	0.58	0.51	0.51
	CI	0.66	0.43	0.52	
	CN	0.50	0.53	0.52	
GFSGD (alpha = 0.9)	AD	0.53	0.72	0.61	0.58
	CI	0.74	0.41	0.53	
	CN	0.55	0.59	0.57	
GFSGD (alpha = 1.0)	AD	0.78	0.84	0.81	0.81
	CI	0.91	0.76	0.83	
	CN	0.77	0.84	0.80	
GFSGD (alpha = 1.1)	AD	0.92	0.98	0.95	0.95
	CI	1.00	0.91	0.95	
	CN	0.94	0.96	0.95	
GFSGD (alpha = 1.2)	AD	0.94	0.95	0.95	0.94
	CI	0.91	0.95	0.93	
	CN	0.96	0.91	0.93	
GFSGD (alpha = 1.3)	AD	0.93	0.99	0.96	0.97
	CI	0.98	0.97	0.97	
	CN	0.99	0.94	0.96	
GFSGD (alpha = 1.4)	AD	0.97	1.00	0.98	0.98
	CI	0.99	0.97	0.98	
	CN	0.99	0.98	0.99	
**GFSGD (alpha = 1.5)**	**AD**	**0.99**	**1.00**	**0.99**	**0.99**
	**CI**	**1.00**	**0.98**	**0.99**	
	**CN**	**0.99**	**0.99**	**0.99**	
GFSGD (alpha = 1.6)	AD	0.89	0.95	0.92	0.91
	CI	0.92	0.87	0.89	
	CN	0.92	0.91	0.91	

### 3.4 Computational efficiency of the proposed Fr-CNN model

The proposed Fr-CNN model provides good balance between accuracy and computational cost. The computational efficiency of the proposed model over other benchmark models is compared through the following metrics:

• **Architectural Parameter(AP)**:

The AP for the convolutional layers is generally computed as:

(11) $$AP=\left(k_{s}\times C_{i}+1\right)\times C_{o}$$

where $k_{s}$ is the size of the kernel, $C_{i}$ and $C_{o}$ refer to the input and output channels. Whereas, the AP for the dense layers can be expressed as:

(12) $$AP=\left(N_{i}+1\right)\times N_{o}$$

where $N_{i}$ and $N_{o}$ are the input and output features.

• **Model Size (MS):**

The general formula for computing the size of the DL-based models is:

(13) MS=No.ofAP×SizeofEachAP

• **Floating Point Operations (FLOPs):**

Similar to architectural parameters, the FLOPs for convolutional layers and dense layers are computed separately and then summed up at the end for the final value. The FLOPs for the convolutional layers are computed as follows:

(14) $$FLOPs=2\times H_{o}\times W_{o}\times C_{o}\times \left(k_{s}\times C_{i}\right)$$

where $H_{o}\;and\;W_{o}$ are the height and width of the output. Furthermore, the FLOPs for dense layers are computed separately through the below formula:

(15) $$FLOPs=2\times N_{i}\times N_{o}$$

The computational efficiency of the proposed Fr-CNN model and other benchmark models is verified through the above evaluation metrics. The performance comparison of the Fr-CNN model over state-of-the-art models in terms of computational efficiency is given in Table 4.

**Table 4:** Computational efficiency of the proposed Fr-CNN model over benchmark models.

Source	Method	Epochs	AP	MS (MB)	FLOPs
Basheera et al. [31]	Custom 2D-CNN	250	134,268,738	512.2	39,400,000,000
Ben Ahmed et al. [32]	Hippocampal Visual Feature Mechanism	200	31,586,547	126	10,100,000,000
Savas et al. [36]	EffecientNetB3	10	61,100,840	244.4	19,000,000,000
Jain et al. [38]	VGG16	–	145,000,000	200	1,600,000,000
Peng et al. [39]	Kernal Learning	50	138,000,000	528	15,500,000,000
Shanmugam et al. [41]	Transfer Learning via GoogleNet	–	42,000,000	168	24,000,000,000
Illakiya et al. [48]	AHANet	–	182,071,364	668	17,000,000,000
Maganti et al. [21]	ADASYN + TL	100	39,134,696	530.76	11,520,000,000
Sorour et al. [22]	CNN + LSTM	–	41,580,858	165	22,000,000,000
Mehmood et al. [23]	Siamese 4D-AlzNet	100	248,780,802	936	15,760,000,000
Hajamohideen et al. [24]	Siamese CNN with triplet loss	250	138,000,000	528	15,500,000,000
Carcagnì et al. [43]	Deit	100	278,380,000	1126.4	37,970,000,000
Li et al. [44]	Ensemble Learning	150	25,200,000	100	1,900,000,000
**Our Proposed**	**Fr-CNN**	**35**	**18,371,305**	**70.08**	**184,000,000**

### 3.5 Best-fit model and comparison with benchmark models

After tuning the essential hyper parameters, it is depicted that the proposed model produces substantial performance in terms of evaluation metrics with novel GFSGD optimizer having alpha rate of 1.5 with learning rate of 0.001. By exploiting undiscovered and novel GFSGD for Alzheimer's disease classification task, better results with computationally cost-effective solution are provided in the study. With improved attention mechanism and effective mixed pooling strategy in the proposed CNN architecture, it results in efficiently extracting prominent features form the MRI scan, which helps the model to attain the best accuracy for the AD classification task. Table 5, shows the brief comparison of the proposed model with benchmark models using MRI images. From the comparison, it is clearly seen that the proposed model outperforms its counterparts in terms of accuracy for the classification of AD.

**Table 5:** Performance comparison of Proposed Model with benchmark models using ADNI database.

Source	Method	Year	Accuracy (%)
Duc et al. [30]	3D-Deep Learning	2020	86.2
Basheera et al. [31]	Custom 2D-CNN	2020	86.7
Ahmed et al. [32]	Hippocampal Visual Feature Mechanism	2015	87.0
Venugopalan et al. [33]	Multimodal Custom 3D-CNN	2021	88.0
Feng et al. [34]	3D-CNN & Support Vector Machine	2020	92.1
Maqsood et al. [35]	AlexNet	2019	92.8
Savas et al. [36]	EffecientNetB3	2021	92.9
Choi et al. [37]	Ensemble Learning	2020	93.8
Jain et al. [38]	VGG16	2018	95.7
Peng et al. [39]	Kernal Learning	2019	96.1
Hon et al. [40]	Transfer Learning via Inception V4	2017	96.2
Shanmugam et al. [41]	Transfer Learning via GoogleNet	2022	96.3
Ebrahimi et al. [42]	Pre-trained ResNet-18	2021	96.8
Beheshti et al. [45]	Genetic Algorithm	2017	97.4
Raju et al. [46]	3D-CNN	2020	97.7
Noman et al. [47]	Transfer Learning	2023	97.8
Illakiya et al. [48]	AHANet	2023	98.5
Buvaneswari et al. [49]	Kernal PCA	2023	98.7
Chen et al. [20]	ResRepANet	2022	89.2
Maganti et al. [21]	ADASYN + TL	2023	87.0
Sorour et al. [22]	CNN + LSTM	2024	98.6
Mehmood et al. [23]	Siamese 4D-AlzNet	2024	95.4
Hajamohideen et al. [24]	Siamese CNN with triplet loss	2023	91.8
Carcagnì et al. [43]	Deit	2023	76.0
Li et al. [44]	Ensemble Learning	2023	98.6
**Proposed Fr-CNN Model**			**99.0**

## 4 Discussion

**Fig. 20:**
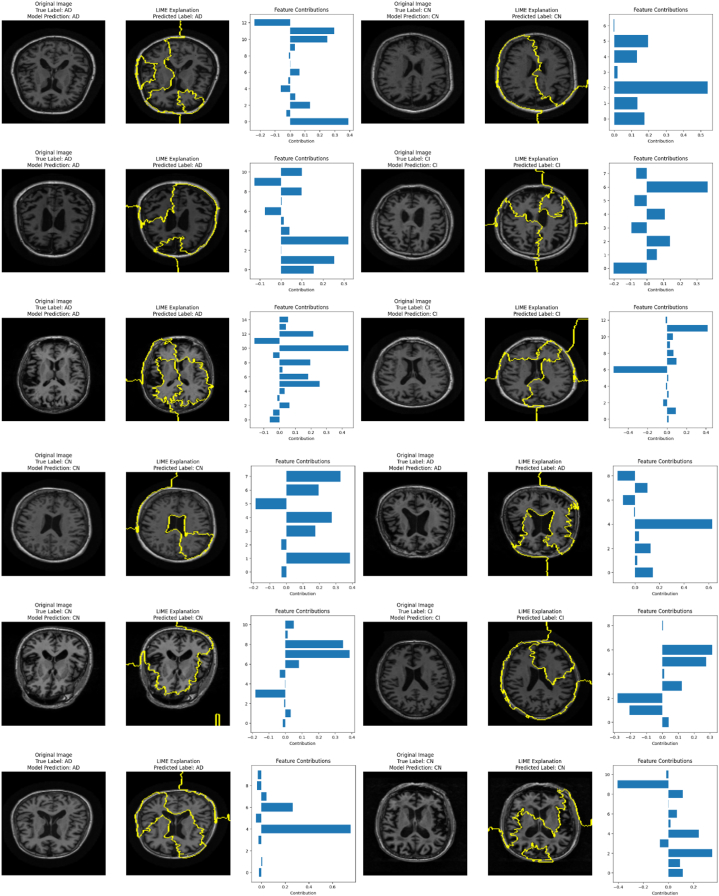
Predictive Capabilities of Proposed Model on Unseen MRI images with Interpretable Explanation.

The proposed model shows remarkable predictive capabilities by accurately classifying unseen MRI scans of different class labels. After rigorous training and evaluation, it is observed that the proposed model is reliable in terms of classifying MRIs among the AD, CI, and CN cases. The proposed Fr-CNN model serves as a noteworthy contribution for accurate detection and classification of AD at premature stages. Transparency in the medical field is essential, so LIME based Explainable AI (XAI) technique is incorporated in the study, for utilizing the capabilities of XAI to propose an interpretable model for explainable classification of AD. LIME encircles the region of most prominent features, on the basis of which the model makes predictions. Seeing the highlighted region, it can be noted which areas of MRIs are important for accurately classifying patients with AD. Some of the interpretable predictions by the proposed model on the unseen images of MRI scans are included in the study and can be visualized in Fig. 20.

## 5 Conclusion

The modification of alpha rates above 1 in standard SGD offers a noteworthy opportunity to tune deep learning model further, which proves to have a positive impact in the study. The proposed Fr-CNN model attained fast convergence speed, when executed with alpha rates above unity. The proposed Fr-CNN model proves to be a notable contribution in AD diagnosis classification. As compared to its counterparts on ADNI benchmark dataset, the improved convergence and accuracy achieved by the proposed model validate the robustness of Fr-CNN over state-of-the-art models. The proposed Fr-CNN model shows substantial performance by achieving an accuracy of 99 % for AD classification task on benchmark dataset through neuroimaging analysis. By utilizing explainable artificial intelligence approach, Fr-CNN model becomes more explicable by providing insightful information about salient features behind the prediction process. The proposed Fr-CNN model's superior convergence and accuracy over benchmark models on standard dataset verify the robustness and adaptability of the proposed work.

### 5.1 Future work

The performance can be further enhanced in the future by employing a more effective strategy with better optimizers and more effective pooling methods. Other XAI techniques can be employed to further enhance the interpretability of the model. This approach is a step towards applying fractional order optimizers in computer vision-based healthcare tasks.

## CRediT authorship contribution statement

**Zeshan Aslam Khan:** Writing – original draft, Conceptualization. **Muhammad Waqar:** Writing – original draft, Methodology. **Naveed Ishtiaq Chaudhary:** Writing – review & editing. **Muhammad Junaid Ali Asif Raja:** Writing – original draft, Methodology. **Saadia Khan:** Visualization, Validation. **Farrukh Aslam Khan:** Visualization, Project administration. **Iqra Ishtiaq Chaudhary:** Visualization, Validation. **Muhammad Asif Zahoor Raja:** Writing – review & editing.

## Declaration of competing interest

The authors declare that they have no known competing financial interests or personal relationships that could have appeared to influence the work reported in this paper.

## References

[1] Uddin M.Z., Shahriar M.A., Mahamood M.N., Alnajjar F., Pramanik M.I., Ahad M.A.R. (Jan. 2024). Deep learning with image-based autism spectrum disorder analysis: a systematic review. Eng. Appl. Artif. Intell..

[2] Xiang Y., Li T., Ren W., Zhu T., Choo K.K.R. (Nov. 2023). A lightweight privacy-preserving scheme using pixel block mixing for facial image classification in deep learning. Eng. Appl. Artif. Intell..

[3] Tong K., Wu Y. (Jun. 2024). Small object detection using deep feature learning and feature fusion network. Eng. Appl. Artif. Intell..

[4] Yu Y., Zhang Y., Cheng Z., Song Z., Tang C. (Nov. 2023). MCA: multidimensional collaborative attention in deep convolutional neural networks for image recognition. Eng. Appl. Artif. Intell..

[5] Sedeh S.S., Fatemi A., Nematbakhsh M.A. (Nov. 2023). Development and application of an optimal COVID-19 screening scale utilizing an interpretable machine learning algorithm. Eng. Appl. Artif. Intell..

[6] Kansal S., Garg D., Upadhyay A., Mittal S., Talwar G.S. (Nov. 2023). DL-AMPUT-EEG: design and development of the low-cost prosthesis for rehabilitation of upper limb amputees using deep-learning-based techniques. Eng. Appl. Artif. Intell..

[7] Liyanarachchi R., Wijekoon J., Premathilaka M., Vidhanaarachchi S. (Oct. 2023). COVID-19 symptom identification using Deep Learning and hardware emulated systems. Eng. Appl. Artif. Intell..

[8] Chen Q., Ye Q., Zhang W., Li H., Zheng X. (Nov. 2023). TGM-Nets: a deep learning framework for enhanced forecasting of tumor growth by integrating imaging and modeling. Eng. Appl. Artif. Intell..

[9] Fu Z., Li J., Hua Z., Fan L. (Oct. 2023). Deep supervision feature refinement attention network for medical image segmentation. Eng. Appl. Artif. Intell..

[10] Raza A., Tran K.P., Koehl L., Li S. (May 2023). AnoFed: adaptive anomaly detection for digital health using transformer-based federated learning and support vector data description. Eng. Appl. Artif. Intell..

[11] (Mar. 2020). “2020 Alzheimer's disease facts and figures,”. Alzheimer's Dementia.

[12] Lim B.Y. (Jun. 2022). Deep learning model for prediction of progressive mild cognitive impairment to Alzheimer's disease using structural MRI. Front. Aging Neurosci..

[13] Khan Z.A., Chaudhary N.I., Zubair S. (Jun. 2019). Fractional stochastic gradient descent for recommender systems. Electron. Mark..

[14] Khan Z.A., Zubair S., Chaudhary N.I., Raja M.A.Z., Khan F.A., Dedovic N. (Jul. 2020). Design of normalized fractional SGD computing paradigm for recommender systems. Neural Comput. Appl..

[15] Khan Z.A., Zubair S., Alquhayz H., Azeem M., Ditta A. (2019). Design of momentum fractional stochastic gradient descent for recommender systems. IEEE Access.

[16] Wei Y., Kang Y., Yin W., Wang Y. (Mar. 2020). Generalization of the gradient method with fractional order gradient direction. J. Franklin Inst..

[17] Janghel R.R., Rathore Y.K. (Aug. 2021). Deep convolution neural network based system for early diagnosis of Alzheimer's disease. IRBM.

[18] Sarraf S., Tofighi G., Org S. (Jul. 2016). Classification of Alzheimer's disease structural MRI data by deep learning convolutional neural networks. https://arxiv.org/abs/1607.06583v2.

[19] Wu H., Luo J., Lu X., Zeng Y. (Jul. 2022). 3D transfer learning network for classification of Alzheimer's disease with MRI. International Journal of Machine Learning and Cybernetics.

[20] Chen Z. (Aug. 2022). A new classification network for diagnosing Alzheimer's disease in class-imbalance MRI datasets. Front. Neurosci..

[21] Maganti S., S. H. A, S. R. (Dec. 2023). Deep transfer learning models for Alzheimer's disease classification using MRI images. International Journal of Intelligent Systems and Applications in Engineering.

[22] Sorour S.E., El-Mageed A.A.A., Albarrak K.M., Alnaim A.K., Wafa A.A., El-Shafeiy E. (Feb. 2024). Classification of Alzheimer's disease using MRI data based on Deep Learning Techniques. Journal of King Saud University - Computer and Information Sciences.

[23] Mehmood A., Shahid F., Khan R., Ibrahim M.M., Zheng Z. (May 2024). Utilizing siamese 4D-AlzNet and transfer learning to identify stages of Alzheimer's disease. Neuroscience.

[24] Hajamohideen F. (Dec. 2023). Four-way classification of Alzheimer's disease using deep Siamese convolutional neural network with triplet-loss function. Brain Inform.

[25] Al-Khuzaie F.E.K., Bayat O., Duru A.D. (2021). Diagnosis of alzheimer disease using 2D MRI slices by convolutional neural network. Appl. Bionics Biomech..

[26] Safi M.S., Safi S.M.M. (Mar. 2021). Early detection of Alzheimer's disease from EEG signals using Hjorth parameters. Biomed. Signal Process Control.

[27] Lu X., Wu H., Zeng Y. (Nov. 2019). Classification of Alzheimer's disease in MobileNet. J Phys Conf Ser.

[28] Antony F., Anita H.B., George J.A. (2023). Classification on Alzheimer's disease MRI images with VGG-16 and VGG-19. Smart Innovation, Systems and Technologies.

[29] Mujahid M., Rehman A., Alam T., Alamri F.S., Fati S.M., Saba T. (Jul. 2023). An efficient ensemble approach for Alzheimer's disease detection using an adaptive synthetic technique and deep learning. Diagnostics 2023.

[30] Duc N.T., Ryu S., Qureshi M.N.I., Choi M., Lee K.H., Lee B. (Jan. 2020). 3D-Deep learning based automatic diagnosis of Alzheimer's disease with joint MMSE prediction using resting-state fMRI. Neuroinformatics.

[31] Basheera S., Satya Sai Ram M. (Apr. 2020). A novel CNN based Alzheimer's disease classification using hybrid enhanced ICA segmented gray matter of MRI. Comput. Med. Imag. Graph..

[32] Ben Ahmed O., Benois-Pineau J., Allard M., Ben Amar C., Catheline G. (Feb. 2015). Classification of Alzheimer's disease subjects from MRI using hippocampal visual features. Multimed. Tool. Appl..

[33] Venugopalan J., Tong L., Hassanzadeh H.R., Wang M.D. (Feb. 2021). Multimodal deep learning models for early detection of Alzheimer's disease stage. Scientific Reports 2021.

[34] Feng W. (May 2020).

[35] Maqsood M. (Jun. 2019). Transfer learning assisted classification and detection of Alzheimer's disease stages using 3D MRI scans. Sensors 2019.

[36] Savaş S. (Feb. 2022). Detecting the stages of Alzheimer's disease with pre-trained deep learning architectures. Arabian J. Sci. Eng..

[37] Choi J.Y., Lee B. (2020). Combining of multiple deep networks via ensemble generalization loss, based on MRI images, for Alzheimer's disease classification. IEEE Signal Process. Lett..

[38] Jain R., Jain N., Aggarwal A., Hemanth D.J. (Oct. 2019). Convolutional neural network based Alzheimer's disease classification from magnetic resonance brain images. Cogn Syst Res.

[39] Peng J., Zhu X., Wang Y., An L., Shen D. (Apr. 2019). Structured sparsity regularized multiple kernel learning for Alzheimer's disease diagnosis. Pattern Recognit.

[40] Hon M., Khan N.M. (2017-January). Proceedings - 2017 IEEE International Conference on Bioinformatics and Biomedicine, BIBM 2017.

[41] Shanmugam J.V., Duraisamy B., Simon B.C., Bhaskaran P. (Jan. 2022). Alzheimer's disease classification using pre-trained deep networks. Biomed. Signal Process Control.

[42] Convolutional neural networks for Alzheimer's disease detection on MRI images. https://www.spiedigitallibrary.org/journals/journal-of-medical-imaging/volume-8/issue-2/024503/Convolutional-neural-networks-for-Alzheimers-disease-detection-on-MRI-images/10.1117/1.JMI.8.2.024503.full.

[43] Carcagnì P., Leo M., Del Coco M., Distante C., De Salve A. (Feb. 2023). Convolution neural networks and self-attention learners for alzheimer dementia diagnosis from brain MRI. Sensors 2023.

[44] Li M., Jiang Y., Li X., Yin S., Luo H. (Jan. 2023). Ensemble of convolutional neural networks and multilayer perceptron for the diagnosis of mild cognitive impairment and Alzheimer's disease. Med. Phys..

[45] Beheshti I., Demirel H., Matsuda H. (Apr. 2017). Classification of Alzheimer's disease and prediction of mild cognitive impairment-to-Alzheimer’s conversion from structural magnetic resource imaging using feature ranking and a genetic algorithm. Comput. Biol. Med..

[46] Raju M., Gopi V.P., Anitha V.S., Wahid K.A. (Dec. 2020). Multi-class diagnosis of Alzheimer's disease using cascaded three dimensional-convolutional neural network. Phys Eng Sci Med.

[47] Raza N., Naseer A., Tamoor M., Zafar K. (Feb. 2023). Alzheimer disease classification through transfer learning approach. Diagnostics 2023.

[48] Illakiya T., Ramamurthy K., Siddharth M.V., Mishra R., Udainiya A. (Jun. 2023). AHANet: adaptive hybrid attention network for Alzheimer's disease classification using brain magnetic resonance imaging. Bioengineering 2023.

[49] Buvaneswari P., Gayathri R. (Nov. 2023). Detection and Classification of Alzheimer's disease from cognitive impairment with resting-state fMRI. Neural Comput. Appl..

[50] Kwak K., Niethammer M., Giovanello K.S., Styner M., Dayan E. (Jan. 2022). Differential role for hippocampal subfields in Alzheimer's disease progression revealed with deep learning. Cerebr. Cortex.

[51] Li F., Liu M. (Jul. 2019). A hybrid convolutional and recurrent neural network for Hippocampus analysis in Alzheimer's disease. J. Neurosci. Methods.

[52] Ali E.H., Sadek S., Makki Z.F. (2023). ICCA 2023 - 2023 5th International Conference on Computer and Applications, Proceedings.

[53] Mazhar T., Haq I., Ditta A., Mohsan S.A.H., Rehman F., Zafar I., Gansau J.A., Goh L.P.W. (Feb. 2023). The role of machine learning and deep learning approaches for the detection of skin cancer. Healthcare.

[54] Haq I., Mazhar T., Malik M.A., Kamal M.M., Ullah I., Kim T., Hamdi M., Hamam H. (2022). Lung nodules localization and report analysis from computerized tomography (CT) scan using a novel machine learning approach. Appl. Sci..

[55] Haq I., Mazhar T., Asif R.N., Ghadi Y.Y., Ullah N., Khan M.A., Al-Rasheed A. (Feb. 2024). YOLO and residual network for colorectal cancer cell detection and counting. Heliyon.

[56] Saqib S.M., Iqbal M., Asghar M.Z., Mazhar T., Almogren A., Rehman A.U., Hamam H. (2024). Cataract and glaucoma detection based on Transfer Learning using MobileNet. Heliyon.

[57] Guo L., Wang Y., Liu H., Li C., Zhao J., Chu H. (2023). ON iterative positive solutions for a class of singular infinite-point P-laplacian fractional differential equation with singular source terms. Journal of Applied Analysis and Computation.

[58] Zhang X., Alahmadi D. (Jul. 2022). Study on the maximum value of flight distance based on the fractional differential equation for calculating the best path of shot put. Applied Mathematics and Nonlinear Sciences.

[59] Qin Y., Basheri M., Omer R.E.E. (Jan. 2022). Energy-saving technology of BIM green buildings using fractional differential equation. Applied Mathematics and Nonlinear Sciences.

[60] Atangana A., Aguilar J.F.G., Kolade M.O., Hristov J.Y. (2020). Editorial: fractional differential and integral operators with non-singular and non-local kernel with application to nonlinear dynamical systems. Chaos, Solit. Fractals.

[61] Atangana A., Gómez-Aguilar J.F. (Sep. 2018). Fractional derivatives with no-index law property: application to chaos and statistics. Chaos, Solit. Fractals.

[62] Atangana A. (Sep. 2017). Fractal-fractional differentiation and integration: connecting fractal calculus and fractional calculus to predict complex system. Chaos, Solit. Fractals.

[63] Mukhtar R., Chang C.Y., Raja M.A.Z., Chaudhary N.I., Shu C.M. (Mar. 2024). Novel nonlinear fractional order Parkinson's disease model for brain electrical activity rhythms: intelligent adaptive Bayesian networks. Chaos, Solit. Fractals.

[64] Wen C., Yang J. (Nov. 2019). Complexity evolution of chaotic financial systems based on fractional calculus. Chaos, Solit. Fractals.

[65] Yang Y., Qi Q., Hu J., Dai J., Yang C. (Oct. 2023). Adaptive Fault-tolerant control for consensus of nonlinear fractional-order multi-agent systems with diffusion. Fractal and Fractional 2023.

[66] Zhang Y., Sun H.G., Stowell H.H., Zayernouri M., Hansen S.E. (Sep. 2017). A review of applications of fractional calculus in Earth system dynamics. Chaos, Solit. Fractals.

[67] Liu Z., Ding Y., Liu C., Zhao C. (Feb. 2020). Existence and uniqueness of solutions for singular fractional differential equation boundary value problem with p-Laplacian. Advances in Difference Equations 2020.

[68] Zhao Y., Sun Y., Liu Z., Wang Y. (2019). Solvability for boundary value problems of nonlinear fractional differential equations with mixed perturbations of the second type. journal/Math AIMS Mathematics.

[69] Araz S.İ. (Jan. 2020). Numerical analysis of a new volterra integro-differential equation involving fractal-fractional operators. Chaos, Solit. Fractals.

[70] Wen L., Liu H., Chen J., Fakieh B., Shorman S.M. (Jan. 2022). Fractional linear regression equation in agricultural disaster assessment model based on geographic information system analysis technology. Applied Mathematics and Nonlinear Sciences.

[71] Shah S.M., Samar R., Khan N.M., Raja M.A.Z. (Apr. 2017). Design of fractional-order variants of complex LMS and NLMS algorithms for adaptive channel equalization. Nonlinear Dynam..

[72] Pu Y.F., Yi Z., Zhou J.L. (Oct. 2017). Fractional hopfield neural networks: fractional dynamic associative recurrent neural networks. IEEE Trans Neural Netw Learn Syst.

[73] Cheng S., Wei Y., Sheng D., Chen Y., Wang Y. (Jan. 2018). Identification for Hammerstein nonlinear ARMAX systems based on multi-innovation fractional order stochastic gradient. Signal Process..

[74] Chaudhary N.I., Aslam khan Z., Zubair S., Raja M.A.Z., Dedovic N. (Feb. 2019). Normalized fractional adaptive methods for nonlinear control autoregressive systems. Appl. Math. Model..

[75] Jia T., Chen X., He L., Zhao F., Qiu J. (Sep. 2022). Finite-Time synchronization of uncertain fractional-order delayed memristive neural networks via adaptive sliding mode control and its application. Fractal and Fractional 2022.

[76] Xing R., Xiao M., Zhang Y., Qiu J. (Feb. 2022). Stability and hopf bifurcation analysis of an (n + m)-neuron double-ring neural network model with multiple time delays. J. Syst. Sci. Complex..

[77] Xu C. (Apr. 2024). New results on bifurcation for fractional-order octonion-valued neural networks involving delays. Netw. Comput. Neural Syst..

[78] Khan M.B., Santos-García G., Noor M.A., Soliman M.S. (Nov. 2022). Some new concepts related to fuzzy fractional calculus for up and down convex fuzzy-number valued functions and inequalities. Chaos, Solit. Fractals.

[79] Li X., Ma W., Bao X. (Mar. 2024). Generalized fractional calculus on time scales based on the generalized Laplace transform. Chaos, Solit. Fractals.

[80] Herrera-Alcántara O., Castelán-Aguilar J.R. (Jun. 2023). Fractional gradient optimizers for PyTorch: enhancing gan and bert. Fractal and Fractional 2023.

[81] Khan Z.A., Chaudhary N.I., Raja M.A.Z. (Jul. 2022). Generalized fractional strategy for recommender systems with chaotic ratings behavior. Chaos, Solit. Fractals.

[82] Wei Y., Chen Y.Q., Gao Q., Wang Y. (Nov. 2019). Proceedings - 2019 Chinese Automation Congress.

[83] Chen Y., Gao Q., Wei Y., Wang Y. (Dec. 2017). Study on fractional order gradient methods. Appl. Math. Comput..

[84] Wei Y., Chen Y., Cheng S., Wang Y. (Dec. 2017). A note on short memory principle of fractional calculus. Fract Calc Appl Anal.

[85] Wei Y., Kang Y., Yin W., Wang Y. (Mar. 2020). Generalization of the gradient method with fractional order gradient direction. J. Franklin Inst..

[86] Jack C.R. (Apr. 2008). The Alzheimer's disease neuroimaging initiative (ADNI): MRI methods. J. Magn. Reson. Imag..

